# Health Disparities in Pharmacy Practice Within the Community: Let's Brainstorm for Solutions

**DOI:** 10.3389/fpubh.2022.847696

**Published:** 2022-04-08

**Authors:** Keri Hurley-Kim, Jacqueise Unonu, Cheryl Wisseh, Christine Cadiz, Erin Knox, Aya F. Ozaki, Alexandre Chan

**Affiliations:** ^1^Department of Clinical Pharmacy Practice, School of Pharmacy & Pharmaceutical Sciences, University of California, Irvine, Irvine, CA, United States; ^2^Department of Clinical and Administrative Pharmacy Sciences, College of Pharmacy, Howard University, Washington, DC, United States

**Keywords:** health disparities, vaccinations, pharmacy desert, pharmacy practice, pharmacists, community

## Abstract

Health disparity is defined as a type of health difference that is closely linked with social, economic and/or environmental disadvantage. Over the past two decades, major efforts have been undertaken to mitigate health disparities and promote health equity in the United States. Within pharmacy practice, health disparities have also been identified to play a role in influencing pharmacists' practice across various clinical settings. However, well-characterized solutions to address such disparities, particularly within pharmacy practice, are lacking in the literature. Recognizing that a significant amount of work will be necessary to reduce or eliminate health disparities, the University of California, Irvine (UCI) School of Pharmacy and Pharmaceutical Sciences held a webinar in June 2021 to explore pertinent issues related to this topic. During the session, participants were given the opportunity to propose and discuss innovative solutions to overcome health disparities in pharmacy practice. The goal of this perspective article is to distill the essence of the presentations and discussions from this interactive session, and to synthesize ideas for practical solutions that can be translated to practice to address this public health problem.

## Introduction

Health disparities are defined as preventable differences in health indicators and outcomes that are closely linked with racial/ethnic, social, economic, or environmental disadvantage (1). They manifest as significant discrepancies in the rate of disease prevalence, incidence, morbidity, mortality, or survival of a marginalized population when compared to the health status of the majority population (2). According to the World Health Organization (WHO), “equity is the absence of unfair, avoidable, or remediable differences among groups of people, whether those groups are defined socially, economically, demographically, geographically or by other dimensions of inequality (i.e., sex, gender, race, ethnicity, disability, or sexual orientation).” Thus, health equity would be defined as the absence of unfair and avoidable or remediable differences in health among population groups that have been defined socially, economically, demographically, or geographically (3). As health inequities are rooted in structural inequity and are systematic differences in the health status of different population groups, health equity can only be achieved when policies and practices that maintain the inequitable distribution of social, economic, and environmental determinants of health are removed and/or reimagined through an equity and justice lens. In short, achieving health equity requires a multifaceted approach to reduce and subsequently eliminate health disparities.

Currently in pharmacy practice, there has been a growing body of literature documenting health disparities and associated social determinants of health (SDOH) common within this discipline (4). Various social determinants have been evaluated as contributing to health disparities in many healthcare settings. Within pharmacy practice, knowledge and perception of health disparities have been identified to play an important role in pharmacists' practice across various clinical settings (5). However, practical solutions to address such disparities are generally lacking in the literature.

On June 4th, 2021, the University of California, Irvine (UCI) School of Pharmacy and Pharmaceutical Sciences hosted a webinar titled “Health Disparities in Pharmacy Practice within the Community.” The online event brought together pharmacy leaders from the university who were organizers of the webinar. Through inviting external guests to participate in case studies sharing and discussions, participants were given opportunities to discuss the barriers and potential driving forces behind implementing health services to overcome health disparities in the community. The goal of this article is to distill the essence of the presentations and discussions from this interactive session, and to synthesize ideas for practical solutions that can be translated to practice to address this public health problem.

### Case Study 1. Identifying Pharmacy Deserts

According to the United States Department of Agriculture and Centers for Disease Control and Prevention, pharmacy deserts are communities with no pharmacy within a one-mile radius or communities with limited vehicle access and no pharmacy within a half mile radius (6). Analyses of Los Angeles County Service Planning Areas (SPAs) based on the USDA and CDC definitions revealed that 571 of 2,323 census tracts were pharmacy deserts and 1,752 were non-deserts. Further analysis with k-means clustering of SDOH population indicators, identified two distinct types of deserts, type 1 and type 2 (7). In total, there were 238 census tracts that were type 1 pharmacy deserts and 333 census tracts that were type 2 pharmacy deserts.

In comparison to type 2 desert residents, type 1 desert residents live in areas in which their SDOH indicators potentially compound the negative effects of being greater than one mile away from a pharmacy (7). Type 1 pharmacy deserts also contain a denser population, more non-Hispanic Black or Hispanic residents, more renters, more people who speak English as their second language, and more residents who might be experiencing linguistic isolation. Socially, type 1 pharmacy deserts occur in areas with less vehicle ownership, fewer health professionals to serve the community, have more residents who are living under the federal poverty level, more residents who experience crimes against property and people, and fewer residents with health insurance. For example, when compared to SPA 6 (South Los Angeles), SPA 5 (West Los Angeles) had almost 5 times as many pharmacies per 1,000 residents even though the population per square mile of SPA 6 was 4 times more than SPA 5. This demonstrates an inequitable distribution of the SDOH within the two types of pharmacy deserts, which drives health disparities along racial, ethnic, and socioeconomic dimensions (7).

The inequitable distribution of social determinants of health in the two types of pharmacy deserts can be attributed to structural inequities which historically and at present, influence population migration in Los Angeles County. Market forces also play a role in the formation of pharmacy deserts through closures and competition for market share. There is a need for more comprehensive analyses of the implications and consequences of pharmacy deserts to population and public health. Residents in pharmacy deserts might benefit greatly from equitable, community-based interventions that can increase access to medications, pharmacy services, and pharmacists (7).

### Case Study 2. Overcoming Vaccination Disparities

Los Angeles County (LAC) in California is the largest county in the United States by population, and has considerable racial, ethnic, and cultural diversity. LAC also has a relatively high rate of poverty (13.4%) with Black and Latinx residents experiencing significant socioeconomic and health-related disparities (8). During the COVID-19 pandemic, the county experienced a massive wave of disease spread beginning in October 2020 through March 2021, with more than 900,000 cases and 16,000 deaths. Consistent with preexisting health- and non-health-related disparities, the distribution of cases was highly uneven in terms of geography, socioeconomic status and race/ethnicity. Latinx and Black residents of South and Central LAC, as well as the Antelope Valley, neighborhoods with higher rates of poverty, were most heavily affected (9, 10).

From December 2020, when COVID-19 vaccines first became available, through July 2021, more than 6.3 million LAC residents received at least one dose of the vaccine. This represented >71% of those who were eligible to receive the vaccine, including nearly 90% of seniors aged 65 and older. Yet vaccinations in the groups that saw the highest rates of COVID infections lagged behind these averages; only about 56% of Latinx and about 47% of Black residents received any vaccine dose by August 1, 2021. At the time of this webinar, many neighborhoods in South LAC and the Antelope Valley had yet to achieve 60% of residents vaccinated (11).

As in many jurisdictions, pharmacists in LAC remain present along every inch of “the last mile” of vaccine distribution in pharmacies, mass vaccination campaigns, clinics, hospitals, and long-term care facilities. The depth and breadth of pharmacists' contributions make us unique among healthcare providers. Throughout, pharmacists have been involved in public health planning, vaccine communication, acquisition and storage, dose preparation, patient education, vaccine administration, management of side effects, and data reporting to governmental agencies. To help address issues of access, pharmacists have also overseen campaigns such as the mass vaccination site at Los Angeles' Dodger Stadium, where nearly a half-million doses were administered in the first half of 2021, and in community health centers that serve as a major source of healthcare for underserved residents, including those who are undocumented. However, it is important to note that many neighborhoods and communities of color continue to experience unequal access to sources of vaccines, including pharmacies (7).

Relatively low vaccination rates and disparate access to healthcare in general in the communities hit hardest by COVID-19 highlight the glaring need to allocate additional, culturally sensitive public health efforts and resources for vaccinating in underserved areas. Moreover, research is needed to investigate the levels and causes of vaccine-related disparities, as well as to determine effective means to address them.

### Case Study 3. Gaining Trust Among Underserved Patients

A patient touch point is any contact spot between a patient and a provider that can occur in any setting (such as hospital, retail, urgent care, ambulatory care clinic, COVID-19 clinic), and that can impact a patient's overall experience and satisfaction with the health care team.

A study of patient touch points published by Shadrav et al. examined the potential benefits of being in contact with patients for at least seven times out of the 12 months of the year (11). These researchers showed that all seven contacts need not be in person, but can include newsletters, phone calls, check-ins, emails, etc. The touch points were used to provide information to patients and to confirm understanding of the instructions or information provided during previous touch points. Each touch point does not have to be limited to one provider and its content can vary, including using time to share information such as general medication education. A benefit of having multiple touch points conducted by multiple providers is that other providers such as a pharmacist can play a key role in managing care without solely relying on physicians for the all touch points. For example, although a pharmacist may have seen a patient 2 or 3 weeks ago, a phone check-in can be scheduled, even when changes to therapy are not necessary. This provides an additional touch point for a patient who may otherwise not be meeting with their physician for several months, which enables a pharmacist to address various issues proactively and creates an opportunity to communicate with the physician provider when needed, and assist in adjusting an action plan prior to the next visit.

The more touch points a patient can have with the healthcare team, the more likely the patient will remain loyal to the care plan, which in turn can improve treatment adherence as well as increase patient engagement in their own health care. A more engaged patient may be more attentive during sessions with the pharmacist and thereby lead to a better understanding of discussion points during counseling. Improving the education of the patient has been linked to increased patient satisfaction which is positively associated with treatment adherence (12). When a patient is engaged, the interaction consists of more than discussing standard information, but also encompasses further assessment of the patient's situation, especially with regard to various SDOH such as financial issues or complex family issues that can hinder health management, which is particularly important for underserved populations. In short, increasing meaningful touch points through the use of pharmacists can be seen as a potential longitudinal approach to building trust among patients, increasing patient engagement, and better creating shared care plans to address patients' specific needs.

## Discussion of Pertinent Issues

**Figure 1:**
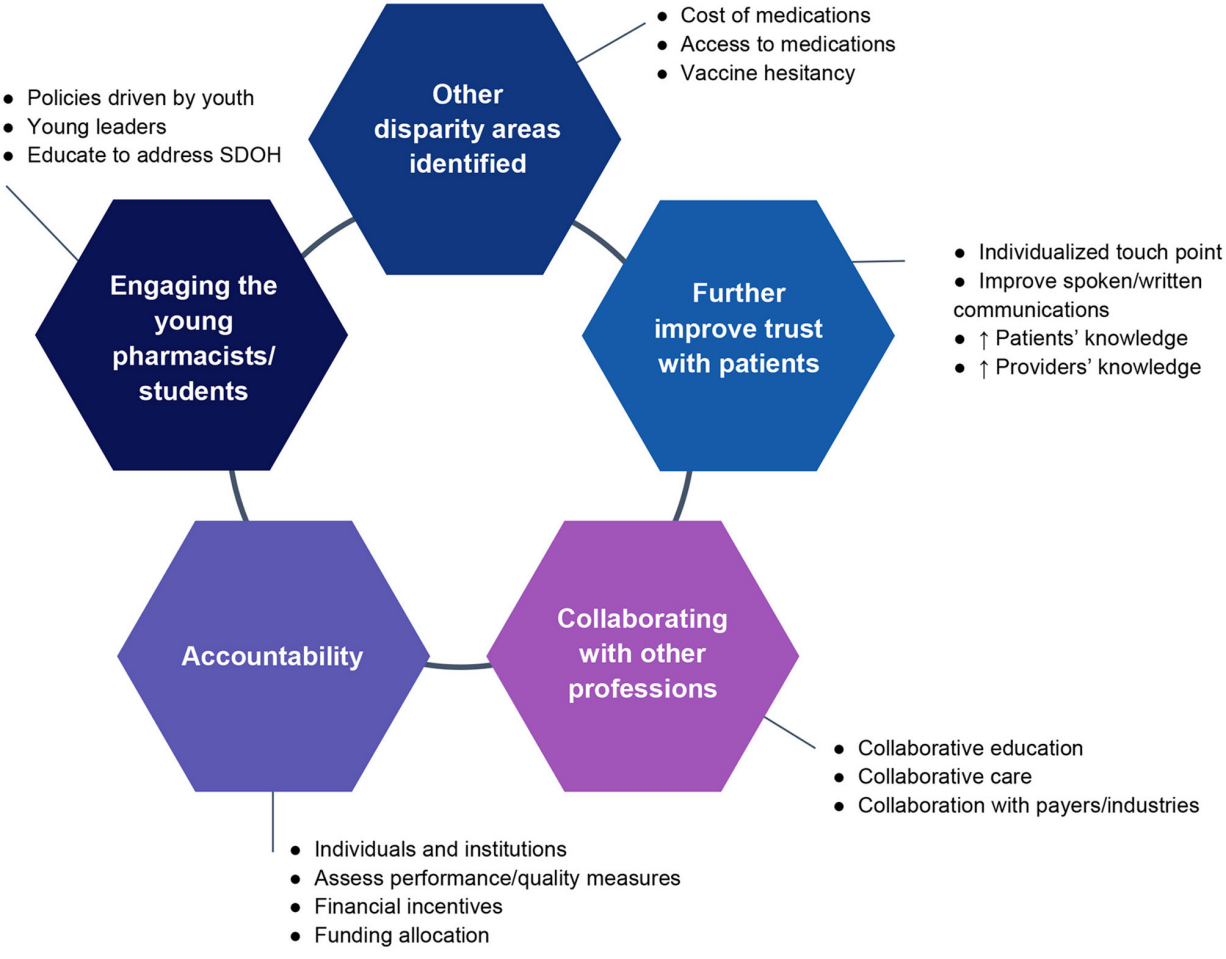
Key issues raised during group discussions. SDOH, social determinants of health.

Following the sharing of these case studies during the webinar, five pertinent issues (Figure 1) surfaced from participant group discussions which were related to the case studies but were not specifically discussed by the speakers, as well as new issues that were introduced by the participants.

### Issue #1. Other Areas of Disparity Were Identified

The cost of medications may affect patients' selection of treatment options. Even when evidence clearly demonstrates superior clinical outcomes with certain medications, patients may inevitably choose cheaper options due to cost considerations or forgo the medication all together. Additionally, patients may compare their medication regimen with those of other patients, thus influencing their decisions about their own treatments. Other disparities can affect access to medications (e.g., access to care, lack of pharmacies in the neighborhood, poor health insurance coverage, language barriers when interpreter services are not available). Given that pharmacists are providers with a wealth of knowledge, bringing pharmaceutical care services including special follow-up or extra touch points to patients with inadequate access services may help address these aforementioned challenges. Vaccine hesitancy is a concerning example of how some of these existing disparities can amplify disparities in COVID-19 vaccine uptake. A practical step to addressing this challenge is to recognize that the onus and the burden of vaccine decision-making and uptake should not be placed solely on the patient who may have low confidence in the vaccines. It is important to understand the historical and socio-cultural context of such concerns, and be aware of vaccine hesitancy among racially, ethnically, and socioeconomically diverse populations. Providers should anticipate, acknowledge, and proactively address these concerns while building trust with the patient.

Medication out-of-pocket costs may also be a significant barrier for socioeconomically disadvantaged patients. By partnerning with pharmaceutical companies, pharmacists are positioned to provide information on sponsored patient assistance programs (PAPs). Pharmaceutical Research and Manufacturers of America (PhRMA) can also partner with pharmacists to provide patient outreach and marketing on the medicine assistance tool. Such tools extend personalized resources for patients to obtain specific medications, often employing PAPs, ultimately improving patient access while enabling PhRMA to meet goals set forth in the organization's commitment to equity (13). Though some concerns exist about utilization of PAPs leading to increased high-cost medication use in order to lower out-of-pocket costs to patients (14), one study has demonstrated that PAPs may have limited impact on prescription drug utilization (15).

### Issue #2. How to Further Improve Trust With Patients

Although multiple touch points are necessary to develop a trusting relationship with patients, the means and frequency should be individualized. Sending emails or newsletters, rather than calling or visiting, might be preferable, for example when providing information regarding medications or adverse drug reactions. However, it is important to note that these communications may not be applicable to patients with poor health literacy and/or language barriers. Other strategies to improve spoken and written communications such as using tools for follow-up patients and creating educational materials are recommended by several health agencies including the Agency for Healthcare Research and Quality (16). The timing of reaching out to patients also should be tailored to individual patients. Instead of providing a large amount of information at once, providing multiple follow-ups based on their need is critical for forming trust. Trust may affect patient behavior or decision-making especially when the situations involve stigmatized conditions or chronic disease states that require complex coordination of management. For example, patients may not trust a government-run mass vaccination clinic vs. clinics or providers with whom they have a previously established a relationship with. It requires time and effort to develop a trusting relationship with patients. Furthermore, there may be a lack of patient knowledge about the roles that pharmacists can play, which leads to the underutilization of these providers. Information on the types of services that pharmacists can provide could increase awareness of their scope of practice and should be widely disseminated to underserved communities. In some situations, it is apparent that pharmacists and other health care professionals had a poor understanding of patient care preferences, particularly for those with different ethnic or racial backgrounds or with different levels of education from their own (17, 18). Within discussions, it was observed that racial and ethnic minority groups often lack trust in the healthcare system, stemming from patients' past experiences, especially given a long history of poor care and disregarded needs among minority individuals.

### Issue #3. Collaborating With Other Professions to Create Solutions

Within the context of a discussion on structural competency, it is important to recognize how social, political, and religious factors in underserved and underrepresented populations impact health (19). One primary issue (and avenue for change) identified was education. Health educators are in a unique position to teach the next generation of health care providers, including pharmacists, beyond what is typically taught in the traditional curriculum. Although pharmacists can play an important role in reducing health disparities, they have historically done so primarily by collaborating with nurses, physicians, and other health care providers. While pharmacists should continue to advocate for collaboration (starting in the educational setting), they should also explore opportunities to expand partnerships by working with social science colleagues and other non-health sector professionals such as anthropologists, policymakers, and lawmakers. These different sector professionals can bring valuable nontraditional perspectives and distinct areas of expertise to help reduce and eliminate health disparities.

### Issue #4. Accountability—How to Successfully Carry Out Proposed Solutions

In order to effectively reduce health disparities, it is important to ensure that individuals and institutions are held accountable for implementing certain changes, or most efforts will fall short. As such, financial incentives and consequences related to reimbursement or accreditation may represent potential means to building accountability. Utilization of metrics including the Healthcare Effectiveness Data and Information Set (HEDIS) or the Health Equity Summary Score (HESS) is useful as performance and quality measures (20, 21). Along this line, funding allocation is another important way to assure that changes are carried out where gaps exist. For example, while mass COVID-19 vaccination clinics did serve a certain percentage of minority groups, this was clearly insufficient. Those who are responsible for mass vaccination clinics should ensure that there is equitable vaccine distribution by prioritizing and implementing more resource-intensive services in areas known to have gaps and low vaccine access and uptake (e.g., in poverty-stricken neighborhoods and rural regions with limited access to health services). Moving forward, funding allocation decisions should be structured so that those who come from underrepresented groups provide input and have a stake in funding streams.

### Issue #5. Importance of Engaging Young Pharmacists

Historically, many social movements related to racial inequities have been led by youth and young adults. For example, much of the drive toward change to anti-racist policies in academic medicine have been driven by medical students and residents. In future efforts to create change, it will be vital to involve more members of groups early in their career trajectories. For decades, the National Pharmaceutical Association and several organizations that include serving marginalized populations as part of their mission have already done work in this area. There is an opportunity to engage young leaders in creating new initiatives and working with faculty and pharmacist champions within professional organizations that conduct outreach in the community. As part of updated standards, the Accreditation Council for Pharmacy Education (ACPE 2016) has emphasized the need for student pharmacists to recognize addressing SDOH as a way to decrease disparities and inequities in quality healthcare (22). In addition, a 2021 ACPE guidance document endorses educational content on health disparities in communities and how pharmacists can serve marginalized populations (23). A curriculum that supports these efforts can foster excitement and enthusiasm in student pharmacists. By applying these educational goals and directives to organizations that train and work with student pharmacists, change could be achieved, harnessing the voice, energy and passion of these young professionals.

## Implications for practice

Our efforts mark the first steps in the search for solutions to reduce and eliminate health disparities in pharmacy practice. While addressing these gaps in care will not occur overnight, the pharmacy profession can begin by prioritizing several actions:

1. Increase and expand collaborative efforts with health and non-health professionals: The pharmacy profession needs to promote multidisciplinary care by expanding team care models to include more pharmacist-led comprehensive medication management programs within health systems and primary care settings including in federally qualified health centers and outpatient settings where pharmacists are integrated into patient-centered medical homes. To foster this type of integrated team care, continued advocacy for provider recognition and policy change in pharmacists' scope of work will be needed. For example, while strategic changes in scope of practice gains for the advanced practice pharmacist designation in several states in the United States have adopted, similar actions are still required in more states to assure that this designation can become relatively uniform nationally and benefit the quality of care for all patients. Additionally, collaboration with non-traditional partners form outside the health care team should be prioritized. The unique perspectives of these professionals, such as social science researchers, public health professionals, policy makers, and lawmakers, can offer important insights and nuanced innovations to address health disparities in pharmacy practice.
2. Identify resources and implement practical solutions: There is a need to identify resources to support this movement, and hold pharmacists accountable for implementing some of the more feasible solutions identified. A specific and timely example may be expansion of programs that support COVID-19 related services in independent community pharmacies. These pharmacies often serve low-income neighborhoods that are under-vaccinated, have less access to testing, and have greater health literacy needs despite initiatives to address them from corporate pharmacies and other healthcare entities. With support from governmental incentive programs, many of these pharmacies can spearhead vaccinations, testing, and patient education in these underserved areas.
3. Educate the next generation of pharmacists, harnessing the voice, energy, and passion of young professionals: The profession should commit to improving the education for the next generation of pharmacists, especially on the importance of mitigating and eliminating health disparities. There is an opportunity to expand and innovate education beyond didactic instruction and to incorporate student pharmacists in this process and in community endeavors that are designed to reduce health disparities.

## Conclusion

Emerging focus areas were identified within pharmacy practice where pharmacists can play a major role to reduce and eliminate health disparities. The issues raised such as access to pharmacies, disparities in vaccination rates, and patient trust in providers have global implications in pharmacy practice. Research studies should be conducted to address these concerns, and ultimately building an evidence base to establish on how pharmacists can play a vital role to help reduce or eliminate structural inequality in healthcare.

## Data Availability Statement

The original contributions presented in the study are included in the article/supplementary material, further inquiries can be directed to the corresponding author/s.

## Author Contributions

KH-K, JU, CW, CC, EK, AFO, and AC interpreted the webinar details and drafted the commentary. All authors read and approved the final manuscript.

## Conflict of Interest

The authors declare that the research was conducted in the absence of any commercial or financial relationships that could be construed as a potential conflict of interest.

## Publisher's Note

All claims expressed in this article are solely those of the authors and do not necessarily represent those of their affiliated organizations, or those of the publisher, the editors and the reviewers. Any product that may be evaluated in this article, or claim that may be made by its manufacturer, is not guaranteed or endorsed by the publisher.
